# Patient engagement and patient-centred care in the management of advanced chronic kidney disease and chronic kidney failure

**DOI:** 10.1186/s40697-014-0024-7

**Published:** 2014-10-21

**Authors:** Robert Allan Bear, Suzanne Stockie

**Affiliations:** Royal Victoria Health Centre, Barrie, ON Canada

**Keywords:** Patient-centred care (PCC), Patient engagement (PE), Nephrology, End-stage Renal Disease (ESRD), Chronic Kidney Disease (CKD), BC Renal Agency (BCRA), Ontario Renal Network (ORN), Patient self-management, Joint decision-making, Advance care planning, Health literacy, Communication

## Abstract

**Purpose:**

The purpose of this article is to review the current status of patient-centred care (PCC) and patient engagement (PE) in the management of patients with advanced chronic kidney disease (CKD) and end-stage renal disease (ESRD), to identify some of the barriers that exist to the achievement of PCC and PE, and to describe how these barriers can be overcome.

**Sources of information:**

The review is based on the professional experience of one of the authors (RB) as a Nephrologist and health care consultant, on the MBA thesis of one of the authors (SS) and on a review of pertinent internet-based information and published literature.

**Findings:**

Evidence exists that, currently, the care of patients with advanced CKD and ESRD is not fully patient-centred or fully supportive of PE. A number of barriers exist, including: conflict with other priorities; lack of training and fear of change; the unequal balance of power between patients and providers; physician culture and behaviour; the fee-for-service model of physician compensation; slow implementation of electronic health records; and, fear of accountability. These barriers can be overcome by committed leadership and the development of an information-based implementation plan. Established Renal Agencies in Canada appear interested in facilitating this work by collaborating in the development of a toolkit of recommended educational resources and preferred implementation practices for use by ESRD Programs.

**Limitations:**

A limitation of this review is the absence of a substantial pre-existing literature on this topic.

**Implications:**

Receiving care that is patient-centred and that promotes PE benefits patients with serious chronic diseases such as advanced CKD and ESRD. Considerable work is required by ESRD Programs to ensure that such care is provided.

Canadian Renal Agencies can play an important role by ensuring that ESRD Programs have access to essential educational material and proven implementation approaches and that implementation successes are celebrated.

In this area, enabling policies are required, as are clinical research studies focusing on the measurement of outcomes.

## Abrégé

**Objectifs:**

L’objet du présent article est, d’une part, de faire le bilan de la situation actuelle sur les soins axés sur le patient et sur la participation du patient dans la prise en charge de patients atteints d’insuffisance rénale chronique (IRC) et d’insuffisance rénale terminale (IRT) et, d’autre part, de définir certains des obstacles à la réalisation des soins axés sur le patient et de la participation du patient, de même que la manière de les contourner.

**Sources d’information:**

Cet article est basé sur l’expérience professionnelle de l’un des auteurs (R. B.) à titre de néphrologue et de conseiller en soins de santé, sur la thèse de MBA de l’un des auteurs (S. S.), de même que sur le survol d’informations pertinentes tirées du Web et de la littérature publiée.

**Conclusions:**

Des preuves démontrent, qu’actuellement, les soins aux patients atteints d’IRC et d’IRT ne sont ni entièrement axés sur le patient ni pleinement favorables à la participation de ces derniers. Il existe plusieurs obstacles, dont la concurrence d’autres priorités, le manque de formation et la peur du changement, l’inégalité des pouvoirs entre les patients et les prestataires de soins, la culture et le comportement des médecins, le modèle des honoraires à l’acte des médecins, la lenteur de la mise en place des dossiers électroniques de santé, et la peur de la responsabilisation. Ces obstacles peuvent être franchis grâce au leadership déterminé et à l’élaboration d’un plan de mise en œuvre basé sur l’information. Les agences de néphrologie semblent souhaiter faciliter ce travail en collaborant à l’élaboration d’une trousse de ressources éducatives recommandées et de pratiques exemplaires de mise en œuvre à l’usage des programmes d’IRT.

**Limites:**

Les limites de cet article constituent l’absence de documentation d’importance sur le sujet.

**Implications:**

Les patients atteints d’IRC et d’IRT bénéficient d’une prestation de soins axés sur le patient et qui favorisent la participation des derniers. Un travail considérable est requis de la part des programmes d’IRT afin d’assurer la prestation de tels soins. Les agences de néphrologie peuvent jouer un rôle important en garantissant l’accès des programmes à du matériel éducationnel essentiel, à des méthodes de mise en œuvre éprouvées, de même que la célébration des réussites en matière de mise en œuvre.

## Why is this review important?

The concept of patient-centred care (PCC) was first advanced in the 1980s, and that of patient engagement (PE) increasingly over the past decade. Receiving care that is patient-centred and that promotes PE improves clinical outcomes, enhances patient and staff satisfaction and may reduce costs. It is of particular importance for patients with serious chronic diseases.

This review is important because it reviews the concepts of PCC and PE, and then focuses these concepts specifically on the clinical areas of advanced CKD and ESRD. To date, this area has received insufficient attention in the Nephrology literature.

## Key messages

The key messages from this review? The Nephrology community has been slow to ensure that patients with advanced CKD and ESRD are receiving care that is optimally patient-centred and that advances PE. The barriers that exist to the implementation of these concepts are described, and a roadmap provided for overcoming these barriers. In Canada, established Renal Agencies such as the BCRA and the ORN appear willing to engage in this task.

## Implications for policy development and research

The need to improve the engagement of patients with advanced CKD and ESRD, so as to provide enhanced PCC, has a number of implications for both policy development and research. Policies designed to promote change in this area through creation of financial and other incentives should be developed and implemented. Research is required to study the comparative effectiveness of different change management strategies across renal programs and, by using specific outcome measures, to develop concrete evidence regarding the benefits of enhanced PE and PCC. Research also is required to determine how best to involve patients and family members in the development of health system research agendas, and the consequences of doing so.

## Introduction

PCC is a current ‘buzz-phrase’ in healthcare. One of us has pointed out that, as a stand-alone concept, the notion of PCC is outmoded, and that achievement of PCC requires PE [1].

The most acknowledged model of PCC was developed by Harvey Picker and the Picker Institute and highlighted in the 1993 book, *Through Patient’s Eyes.* In 2001, the Institute of Medicine emphasized the touchstone importance of patient-centred care and referenced the Picker Principles in its landmark publication: *Crossing the Quality Chasm: A New Health System for the 21st Century* [2].

Subsequently, The Change Foundation, an independent Canadian health policy think tank (www.ChangeFoundation.ca) conducted focus groups with patients to develop a set of Patients’ Principles of Patient- Centred Care. These essentially parallel the Picker Principles, reframing them in patients’ words and emphasizing what patients consider to be of greatest importance.

The Patients’ Principles of Patient-Centred Care, as summarized by Stockie [3], are as follows: *Comprehensive care* - all patient needs, not just some, to be addressed; *Co-ordination of care* - someone is in charge; there is someone to go to who knows the patient and will help the patient navigate the system; *Timeliness* - patients to get care when they need it and, where a sequence of services is required, intervals to be short; *Functioning e-health* - patients to provide their information only once, it to be accessible to those who need it, and patients to have access to their records and the opportunity to add to them; *Clear and reliable communication* - when delivering information, health care providers to ensure it is understood, and explain and clarify as required; messages to be consistent; access to phone and internet consultations to be available; *Convenience* - the need to go to different physical locations for services to be minimized. There should be open access, same day scheduling and no unnecessary barriers to getting the right provider; *Respect* - for their time; their intelligence; the validity of their stories; their feedback about quality and effectiveness; and, their environment, family and care giving partners; *Empathy and understanding* - of their circumstances, fears, hopes, and psychological state; *Time* - to express needs and be heard effectively; *Continuity and stability* - to know and be known; to minimize the number of different care providers; *Fairness* - the amount and timeliness of service to be commensurate with need.

The Patients’ Principles of Patient-Centred Care clearly articulate a set of contemporary patient expectations. And they convey an emotionality that cannot be ignored, emotionality also evident in dialysis patient blogs [4].

About this emotionality, Donald Berwick once said: “PCC is a verbal analgesic disguising real pain” [5], thus suggesting that use of the ‘buzz-phrase’ PCC may engender in some patients a soothing illusion of an improved style of care, but it is just that, a soothing illusion. This is because the principles of PCC are basically a set of prescriptions for optimizing provider team behaviour and do not place sufficient responsibility on patients themselves. On their own, they will not effect real change and reduction in patient pain. Real change requires PE.

PE is: Patients taking an active role in their care, doing what they need to do to ensure that, in their care, the principles of PCC are being met. It has been said that PCC is best recognized when it is absent. On the other hand, PE is best recognized when it’s present.

Engaged patients are comfortable openly expressing their fears and anxieties. They question, insist upon exploring options, and ensure a focus on quality-of-life. Engaged patients talk more and listen less; accordingly, members of the provider team talk less and listen more. Finally, engaged patients want to be accountable.

Studies increasingly suggest that PE, and the PCC it supports, is associated with enhanced patient and provider satisfaction, improved outcomes, and reduced costs [3,6].

## Review

### Evidence that more work is required to advance PCC and ensure patients are optimally engaged

The authors do not believe that most patients receiving care for advanced CKD or ESRD are fully ‘engaged’ or receiving care that is optimally patient-centred. Evidence in support of this belief can be found in the answers to the following questions:

### In most ESRD programs, is there a highly-publicized commitment to PE, and through PE, to PCC? If so, is this commitment accompanied by an action plan?

Despite notional commitments of health care organizations to the concepts of PCC and PE, concrete Action Plans related to them in ESRD Programs are uncommon. To counter this, the Center for Medicare and Medicaid Services (CMS) in the United States recently established a requirement for all dialysis units receiving Medicare funds to develop and implement initiatives designed to advance PE; information regarding the status of these early initiatives can be found on CMS Regional ESRD Network websites, such as that of the ESRD Network of Texas [7]. Canada lacks this type of widespread initiative.

### Do the majority of advanced CKD patients receive care in an interdisciplinary pre-dialysis clinic?

Ensuring that patients with advanced CKD have access to interdisciplinary pre-dialysis care is almost a precondition for PCC, since it is in such environments that patient and family education can best be achieved, and joint decision- making employed regarding such matters as dialysis modality choice or choosing conservative care only. Also, educating patients about the principles and techniques of PE is best accomplished in this non-threatening ambulatory care environment; patients who learn the techniques of PE in a CKD clinic will then take those skills with them when they move to dialysis. However, in the U.S. and, to a lesser extent in Canada, many patients continue to be introduced to dialysis without having received any pre-dialysis care, and the pre-dialysis care of those who do receive it, as judged by late care, lower cumulative care and inconsistent critical period care, may be inadequate and associated with higher one-year mortality [8]. Additionally, education of patients in the principles and techniques of PE is not standard practice within all pre-dialysis clinics.

### Have most ESRD Programs implemented specific initiatives that both address the low literacy skill of some patients and improve the health literacy of all patients?

In the general population, low literacy skill is common. Furthermore, various studies report that, despite the availability of high-quality educational material [9-11], up to one-third of advanced CKD and ESRD patients, irrespective of their literacy skills have low health literacy about their disease [12-18]. Low literacy and health literacy are formidable barriers to achieving both PCC and PE. Low health literacy is associated with sub-optimal decision making, uninformed dialysis modality choice, poor clinical outcomes and non-adherence in hemodialysis [19-21]. Screening tools for low literacy [22] and low health literacy [23] exist, but many ESRD Programs do not feature these, thus limiting remediation opportunities; as a consequence, low literacy and health literacy remain major clinical care issues.

### In most ESRD Programs, do formal educational programs exist in the use of specific tools designed to enhance the communication skills of both providers and patients?

Specific tools and techniques designed to improve communication exist for both patients and healthcare providers [24-27]. In 2012, Schell et al. published a report on NephroTalk, in which a number of these tools were used to enhance the communication skills of Nephrology trainees, with positive results [28]. Despite this example, and others, communication education tools are not routinely employed by kidney programs. This is unfortunate. Studies show that untrained providers, in their interactions with ESRD patients, tend to ignore emotional data and focus on the clinical (28). Teaching communication skills to providers increases their capacity to recognize and respond to emotional issues [29]. Improving communication between providers and patients is a core requirement for both PE and PCC.

### In most ESRD Programs, are educational programs on patient self-management offered to providers and patients?

Patient self-management is a useful tool for advancing PE. Increasing the self-management skills of patients requires education of healthcare providers. Excellent educational materials are available [30-32].

These are ‘early days’ for patient-self management in ESRD Programs, few of which feature specific educational programs on patient self-management for physicians, staff and patients. This is beginning to change [33-36].

### Are processes supporting shared decision-making evident in most ESRD Programs?

Shared decision-making is an essential component of both PCC and PE [37]. However, there has been insufficient attention to ensuring providers possess the skills and tools necessary for the facilitation of shared decision-making. A study of 197 ESRD patients demonstrated a gap in the degree to which they wanted joint decision-making and the degree to which they were afforded it [38]. Morton et al described the usual ‘influencers’ of important decisions such as dialysis modality choice and withdrawal from dialysis and showed that shared decision-making is not common in such circumstances [39]. Various studies on end-of-life care in the advanced CKD and ESRD populations support this view (see below). Shared decision-making requires patient health literacy, augmented communication skills and engaged patients - each of which is far from universally present.

### In most ESRD Programs, are processes related to Advance Planning congruent with a commitment to PE and PCC?

A quick test of the patient-centredness of any health system, hospital or program: the manner in which advance planning, in all of its aspects, is conducted. Holley has observed that in the ESRD patient population: “[advance planning] should be a comprehensive and patient-centred process used by patients and families to strengthen relationships, achieve control over medical care, prepare for death and clarify goals of care [40].” The Renal Physicians Association and the American Society of Nephrology have published guidelines to ensure the above is achieved [41], but much remains to be done. Davison *et al* conducted a survey of American and Canadian Nephrologists, and only 39% self-identified as being well-prepared for the conduct of end-of-life discussions [42]. An abstract presented at the 2013 ASN Annual Meeting revealed that current Nephrology residents are no better trained in palliative care than they were 5 years ago [43]. In a recent study of 138 deaths of patients with ESRD [44], 69% died in hospital (in 80%, their preferred place of death had not been determined). Only 28% had discussed their end-of-life care with a provider in the previous year. In only 64% of the deaths was there a recorded conversation with the patient and/or family about death during the final admission. 36% of the deaths were “unexpected”, although most could have been predicted, and these deaths were of lower quality.

### Does the focus of scientific research in Nephrology suggest that funding agencies and clinical investigators consider projects related to PCC and PE to be research priorities?

An informal review by one of the authors of the 3814 abstracts of work presented orally or by poster during the 2013 American Society of Nephrology Annual Meeting revealed that only 2 of the presentations described patient-centred care programs and only 28 described concrete initiatives with specific outcomes in areas that could be considered related to PE or PCC [45].

Is there evidence that research priorities in Nephrology as defined by funding agencies and clinical researchers are aligned with the research priorities of patients with advanced CKD or ESRD?

There are few data on this topic, but a recent report suggests that patients are interested in research related to matters such as improving communication between providers and patients, optimizing dialysis modality choice processes, management of emotional symptoms, and physical symptom relief [46]. These are not the evident research priorities of funding agencies and clinical investigators (see above).

## Barriers to the achievement of PE and PCC

Why is there so much left to do in achieving PE and PCC in ESRD Programs? A number of barriers exist.

### Conflict with other priorities

The advancement of PCC and PE in an ESRD Program will not occur organically. A commitment must be made by the parent organizations of the ESRD Program and plans, strategies and tactics developed, the implementation of which will require investment of time and resources. It is an unfortunate reality that these implementation needs can be expected to be in constant conflict with other priorities, often considered more urgent.

### Lack of training and fear of change

Few staff, physicians and patients within ESRD Programs have received specific training in tools, techniques and skills specifically related to advancing PE and PCC. Being insufficiently prepared for change can make one more fearful of it.

### The unequal balance of power between providers and patients

Providers within ESRD Programs have more clinical knowledge about CKD and ESRD than their patients, and administrators usually perceive themselves as expert in the design and delivery of health care. ESRD patients, on the other hand, are vulnerable, frightened and often experiencing discomfort, all of which can increase their dependence on their providers and program administrators.

### Physician culture and behaviour

The behaviour of physicians is embedded in their education and socialization. Physicians need to move from ‘doing to’ and ‘doing for’ to ‘doing with’. This is being accomplished to some extent in the U.S., where the majority of physicians is now employed by health systems and hospitals and where there are system incentives related to the advancement of PE and PCC. In Canada, a greater percentage of physicians operate as independent contractors, more accountable to their discipline than to their organization. Both the Institute of Medicine and the Canadian Medical Association have emphasized the importance of patient-centred care. If the requisite culture change is to occur, physicians must be informed, provided with the necessary tools and skills and nurtured. Much remains to be done.

### Fee-for-service physician compensation

The fee-for-service payment model is a barrier to PE and PCC in many ways.

### Slow implementation of electronic health records (EHRs)

Lack of availability of patient-accessible, interactive EHR systems impedes patients from taking an active role in managing their health.

### Fear of accountability

If a health system, hospital or ESRD Program openly engages patients and families, it must be willing to listen and respond to patients’ feedback. This is not always easy.

## Overcoming barriers and advancing PCC and PE in ESRD programs

If an ESRD Program, with the support of the Board and Senior Leadership Team of its parent organization, is committed to ensuring the care it delivers is optimally patient-centred, and promotes PE, how best to proceed? The fundamental challenge is to achieve a change in organizational culture. An organization’s culture is its lifeblood, and it is formed by the deeds of its leaders. If an ESRD Program is to be driven by the notion of patient-centredness, it will be because its medical and nursing and administrative leaders are unflinchingly determined to make it so.

However, committed and determined leadership, while necessary, is not sufficient. Skills must be taught, tools selected and made available, and enabling structures thought about and created. While it is possible for individual ESRD Programs to accomplish these tasks, it is difficult.

In the U.S., the CMS has asked each ESRD Program to design and implement its own approach to the enhancement of PE, and the results of these approaches are being shared [7]. In Canada, preliminary discussions with the Leadership of the BC Renal Agency (BCRA), and the Ontario Renal Network (ORN) reveal a willingness to explore ways to work together to identify a preferred set of educational materials for ESRD Programs seeking to advance PCC and PE. These would include best practices in addressing low patient literacy and health literacy, in educating providers and patients on communication competencies, and in the fostering of patient self- management and joint decision-making skills. The BCRA and ORN also appear interested in identifying best practices related to the creation of enabling roles and structures (e.g. physician champions, patient advisors, multi-stakeholder PCC/PE Advisory Committees) and effective implementation approaches. If Canadian agencies such as the BCRA and ORN (and potentially the Northern Alberta and Southern Alberta Renal Programs) do choose to work collaboratively on the above, they may also choose to undertake the task of ensuring that notable implementation success stories are widely communicated: of patients being integrated into an ESRD Program’s clinical policy development and decision-making processes; of patients providing meaningful input into development of an organization’s research priorities; of the specific outcome measures that are most useful in measuring progress; of examples of improved work environments and enhanced efficiency; of documented enhancement of the patient experience. If this is accomplished, the wide availability of such information will undoubtedly encourage additional Canadian ESRD programs to adopt patient-centredness as their Program Vision, and begin to move toward it. And over time, these Programs will find ways of sharing their experiences and successes, thus contributing to change on a larger scale.

This is an important Canadian challenge and opportunity.

## Outcome measures

If an ESRD Program decides to create and implement an Action Plan intended to foster the development of an organizational culture of patient-centredness, it must establish outcome measures through which progress is tracked and reported upon. Potential outcome measures can be classified as *structural* and *functional. Structural* outcome measures include: The Organization’s Board and Senior Leadership Team are informed and committed; A physician champion has been identified; Trained patient advisors are in place; A multi-stakeholder Patient and Family Advisory Council is at work; A Program self-assessment on patient engagement has been completed; A preferred organizational culture has been identified; An Implementation Action Plan exists; There is an implementation toolkit for patients, staff and physicians. *Functional* outcome measures include: There are ongoing ESRD Program initiatives in: literacy and health literacy; communication skills; patient self- management; and, joint decision-making; Patient advisors are involved in policy development on clinical matters; Care plans are developed in partnership with patients; Data demonstrate improved dialysis patient adherence; Data reveal that a contemporary Advance Planning Program is resulting in reduced hospital deaths, increased home deaths, and increased hospice care; Patients are providing input on the Organization’s research agenda; Operating costs are reduced; Staff satisfaction is increased; staff turnover is reduced; The are measurable improvements in the ‘Patient Experience’; The ESRD Program’s implementation successes are being celebrated and shared.

## Conclusion

Chronic kidney failure is a serious disease. Its pathophysiology is complex, and leads to an annual dialysis patient mortality rate that approaches 20%. It is both understandable and appropriate, then, that tremendous scientific effort has and continues to be placed on unraveling the secrets of this pathophysiology. As a result of this effort, the care provided to patients with CKD and ESRD continues to improve.

However, this scientific focus also engenders a risk: that the more human aspects of this disease do not receive the attention they deserve. Patients with advanced CKD and ESRD - along with their families and loved-ones - confront many challenges beyond those related to co-morbidities and to the pathophysiology of their disease. They must become ‘health literate’ about the nature of CKD and its consequences; difficult treatment choices must be made - including consideration of only conservative care; lifestyle adjustments - at times including those related to employment and financial security - will be required; the natural history and prognosis of advanced CKD and ESRD must be understood, confronted and accepted, and life expectations must be adjusted.

While confronting all of the above, many patients describe themselves as suffering. Many, as they approach the end- of-life, say they have lost dignity, feel a sense of hopelessness and wish for death [47], and many would prefer that greater attention be paid to their symptoms as opposed to their outcomes. They have “an aversion to worsening symptoms with indifference to survival” [48].

It is essential that ESRD Programs respond to all of the needs of their patients - physical, emotional and psychological. Ensuring their patients are engaged and that their care is patient-centred will assist in this. Barriers exist, but these barriers can be overcome through commitment and planning. As this happens:

*“It will be better for people…*

*It will be better for those who care for people.*

*We will wonder why we were so afraid of change for so long.*

*We will regret the harm that could have been prevented had we changed sooner.*

*We will regret the good not done because we didn’t change sooner and on a larger scale.”*

– Stephen Lewis, 2012 (As referenced by Stockie [3]).

## Abbreviations

CKD: Chronic kidney disease
ESRD: End-stage renal disease
PCC: Patient-centred care
PE: Patient engagement
CMS: Center for Medicare and Medicaid Services
